# Balanced Solutions Versus Saline to Reduce AKI: A #NephJC Editorial on the BaSICS Trial

**DOI:** 10.1016/j.xkme.2022.100472

**Published:** 2022-04-29

**Authors:** Mythri Shankar, Carlo Trinidad, Elliot Koranteng Tannor, Swapnil Hiremath, Joel M. Topf

**Affiliations:** 1 Department of Nephrology, Institute of Nephrourology, Bengaluru, Karnataka, India; 2 Department of Medicine, Dagupan Doctors Villaflor Memorial Hospital, Pangasinan, Philippines; 3 Department of Medicine, Kwame Nkrumah University of Science and Technology, Kumasi, Ghana; 4 Department of Medicine, University of Ottawa, Ottawa, ON, Canada; 5 Department of Medicine, Oakland University William Beaumont School of Medicine, Auburn Hills, Michigan

*#NephJC is a recurring twitter-based journal club. #NephJC editorials highlight the discussed article and summarize key points from the NephJC TweetChat.*

Medicine has adopted the use of intravenous fluids as a foundational treatment some 185 years ago. Buffered saline solutions were first used in the resuscitation of patients during the London cholera epidemic of 1832.^1^ Intravenous fluids are still the primary intervention to treat shock. Despite this long history, fundamental questions regarding the content, timing, rate, and amount of fluid remain unanswered.^2^ In this editorial, we discuss the literature around the use of balanced solutions and kidney injury. Balanced solutions are variously referred to in the literature as “buffered saline solutions,” “balanced multielectolyte solutions,” “chloride-restricted solutions,” or “balanced crystalloids” and have in common a lower chloride concentration (typically 98-110 mmol/L), addition of a buffer (lactate, gluconate, and/or acetate), and a small amount of other electrolytes (potassium, calcium, or magnesium).

Until recently, the resuscitation fluid of choice, especially for internists, was normal saline. It is cheap, widely available, and familiar. In 2012, Yunos et al^3^ conducted a prospective, open-label, sequential-period pilot study in 760 patients admitted to a multidisciplinary intensive care unit (ICU), comparing a chloride-restricted resuscitation strategy with a chloride-liberal resuscitation strategy. During the 6-month control period, all patients admitted to the ICU received normal saline, which was followed by a phase-out period of 6 months. Following the phase-out period, all ICU patients received chloride-restricted fluids (Plasma-Lyte 148, Hartmann’s solution, chloride-poor 20% albumin) for the next 6 months. They reported a significant reduction in acute kidney injury (AKI) and requirement of kidney replacement therapy (KRT) with a chloride-restrictive strategy.^3^ Although the article reports AKI as the primary outcome, the initial outcome according to ClinicalTrials.gov was the change in the mean base excess during hospitalization.^4^

**Figure 1:**
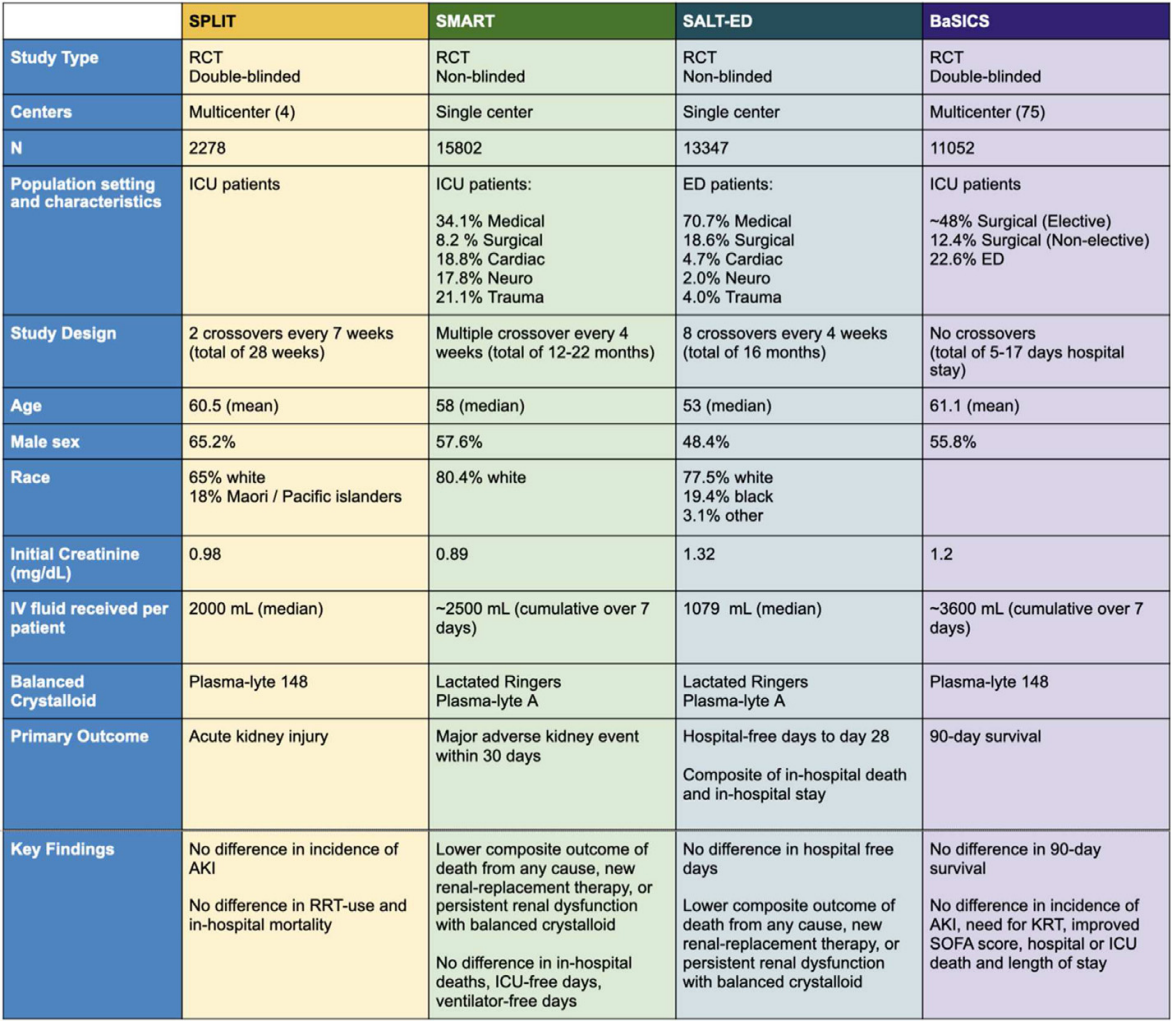
A comparative table of important studies published.

In 2015, Young et al^5^ published the SPLIT (0.9% Saline vs Plasma-Lyte 148 for Intensive Care Unit Fluid Therapy) trial, a cluster-randomized trial of normal saline versus Plasma-Lyte conducted in 4 ICUs in New Zealand. They did not find any difference in AKI or the need for KRT.^5^ Then, in 2018, the SMART (Isotonic Solutions and Major Adverse Renal Events Trial) and SALT-ED (Saline Against Lactated Ringer’s or Plasma-Lyte in the Emergency Department) pragmatic trials were published.^6^^,^^7^ Both these were single-center, open-label, cluster-randomized trials, in which the intravenous solutions were alternated every month. The SALT-ED trial was conducted in the emergency department, and the SMART trial was conducted in ICUs. The SALT-ED trial reported no difference in hospital-free days between the groups (balanced crystalloid vs normal saline). However, the secondary outcome, which was major adverse kidney events (a composite of death because of any cause, initiation of KRT, and persistent kidney dysfunction, with the latter defined as an inability to recover 50% of the baseline estimated glomerular filtration rate when evaluated up to 90 days after discharge), was lower in the balanced crystalloid group than in the saline group (odds ratio, 0.82; 95% confidence interval, 0.70-0.95). The SMART trial similarly showed that balanced crystalloids reduced major adverse kidney events than the saline group (odds ratio, 0.91; 95% confidence interval, 0.84-0.99). Although positive, the SMART and SALT-ED trials were neither blinded nor randomized at the individual patient level, and some questioned the effect sizes given the small amount of fluid administered (see Fig 1 for a comparison of all major studies in this field).

### The BaSICS Trial

Balanced Solutions in Intensive Care Study (BaSICS) was a multicenter, randomized controlled trial with a 2 × 2 factorial design comparing balanced crystalloids to normal saline and slow versus fast infusion among critically ill patients.^8^ It was conducted in 75 Brazilian ICUs from May 2017 to March 2020. Physicians, patients, investigators, and outcome assessors were all blinded. The detailed eligibility criteria are provided in Box 1. Briefly, ICU patients with hypotension or on pressors and not already with established AKI on KRT or those with severe hyponatremia or hypernatremia were included in the trial.

Box 1 **Eligibility Criteria for the BaSICS Trial**

*Inclusion criteria*

ICU patients meeting at least 1 of the following criteria:

• Age greater than 65 y
• Hypotension (MAP < 65 mm Hg, SBP < 90 mm Hg, or vasopressor use)
• Sepsis
• Requiring mechanical or noninvasive ventilation for at least 12 hours
• Oliguria (<0.5 mL/kg/h for ≥3 h) or azotemia (creatinine level of >1.2 mg/dL for women and >1.4 mg/dL for men)
• Liver cirrhosis or acute liver failure

*Exclusion criteria*

• AKI requiring RRT within 6 h of admission
• Severe electrolyte disturbance (serum sodium level ≤ 120 mmol/L or ≥ 160 mmol/L)
• Imminent death within 24 h
• Suspected or confirmed brain death
• On palliative or comfort care
• Previously enrolled in the trial
• Serum potassium level of >5.5 mEq/L (added after second interim analysis)

Abbreviations: AKI, acute kidney injury; BaSICS, Balanced Solutions in Intensive Care Study; ICU, intensive care unit; MAP, mean arterial pressure; RRT, renal replacement therapy; SBP, systolic blood pressure.

The participants were randomized to receive either a balanced solution (Plasma-Lyte 148) or 0.9% saline at 2 different infusion rates (333 mL/h and 999 mL/h). Administration of nonstudy fluids by clinicians was allowed. The primary outcome was the 90-day survival. Secondary outcomes measured were the need for KRT, occurrence of AKI, and Sequential Organ Failure Assessment scores at days 3 and 7. Tertiary outcomes were ICU admission, hospital mortality, and the length of hospital stay.

A total of 5,230 patients were randomly assigned to receive a balanced solution, and 5,290 received 0.9% normal saline solution. The mean age was 61 ± 17 years, and the mean serum creatinine level was 1.2 mg/dL. Almost half of the patients (48.4%) were admitted to the ICU after elective surgery, and 68% received crystalloid fluid bolus before enrollment. Within 90 days, 1,381 (26.4%) patients assigned to the balanced solutions died versus 1,439 (27.2%) patients assigned to the saline solution (*P* = 0.47). There was no significant interaction between the 2 interventions (fluid type and infusion speed; *P* = 0.98) or between groups for the primary outcome. The 90-day mortality rate was significantly higher in patients with traumatic brain injury receiving balanced solution than in those receiving the saline solution (31.3% vs 21.1%; *P* = 0.02). The neurological Sequential Organ Failure Assessment score on day 7 was significantly higher in the balanced crystalloid group than in the saline group. No differences in the occurrence of AKI or need for KRT were observed. ICU admission, hospital mortality, and length of stay were similar between groups. Thus, there was no benefit with balanced solutions compared to normal saline for any clinical outcome or in any subgroup.

### Tweetchat

**Figure 2:**
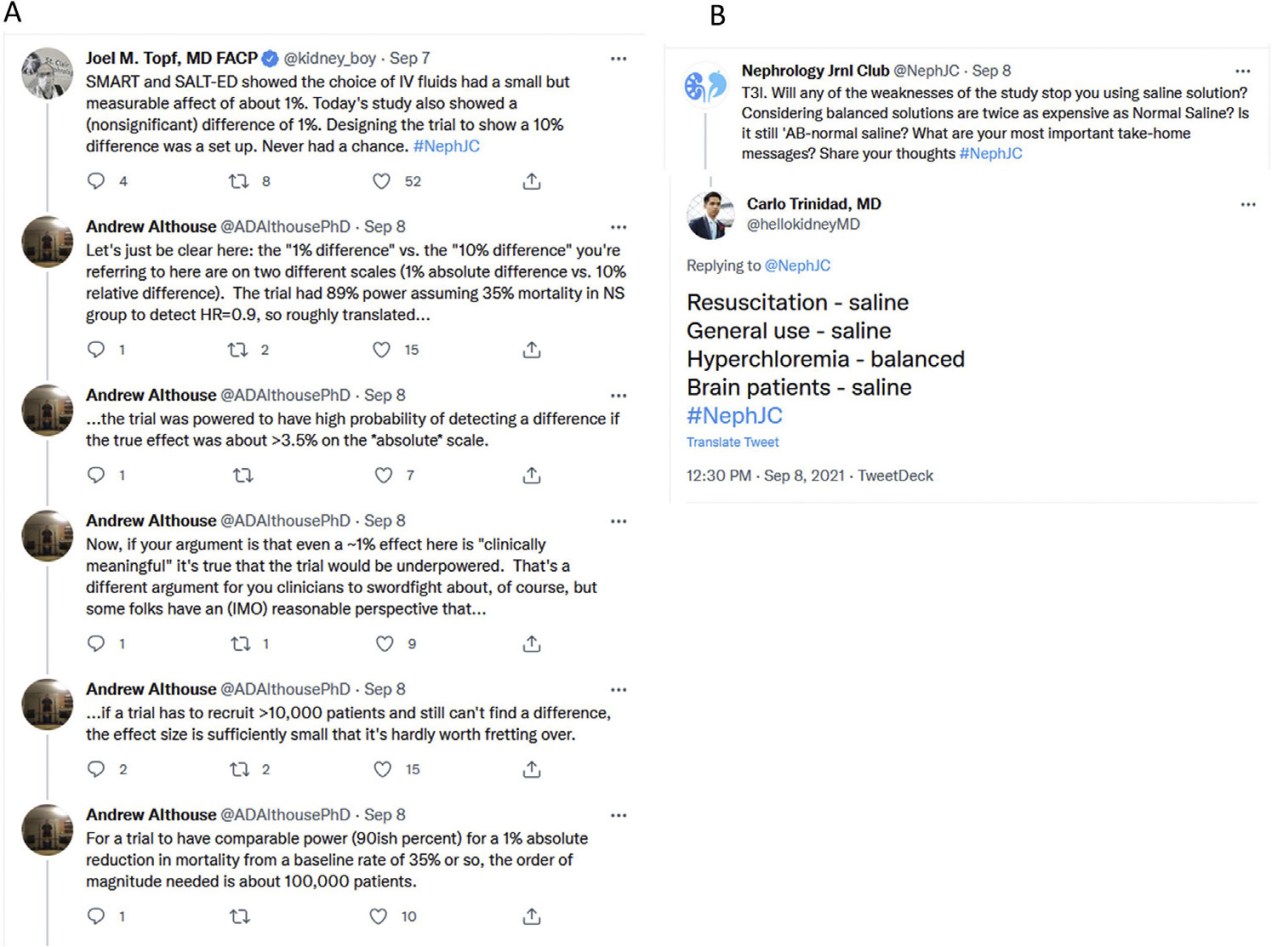
(A) Twitter discussion about choice of outcome, sample size, and power. (B) Twitter discussion of BaSICS trial results changing clinical management choice. BaSICS, Balanced Solutions in Intensive Care Study.

The NephJC Tweetchats on the BaSICS overall had 180 active participants and 795 tweets. We conducted 2 online Twitter polls before the Tweetchat with the question “What is the best intravenous fluid for the ICU patient? Is it Ringers/balanced crystalloid or saline solution?” From 332 responders, the balanced solutions were chosen by just >60% (Fig 2).

During the discussion, it was noted that the SALT-ED and SMART trials had already resulted in a practice change for many practitioners, as reflected in the poll. One big change was the replacement of normal saline solution with lactated Ringer’s solution in the sepsis care bundle.^9^ Those who had not changed practice (and felt vindicated by the BaSICS results) pointed to a number of factors from the SMART and SALT-ED trials, such as the cluster-randomized design, the small amount of fluid administered, and the outcome being driven by a change in creatinine rather than a hard endpoint like death or dialysis. Additionally, the cost of balanced solutions came up, as lactated Ringer’s solution has been reported to cost $2.50 more than saline solution.^10^ However, the author of that article chimed in during the chat that since the time of writing of that review (2019), the cost of lactated Ringer’s solution has fallen to be roughly in line with normal saline solution, although Plasma-Lyte remains more expensive.

The positive findings from the SMART and SALT-ED trials were a reduction in major adverse kidney events (which was the primary outcome in the former trial and the secondary outcome in the latter), whereas the null finding in the BaSICS trial was the lack of difference in 90-day survival, and there was some discussion about whether expecting a 10% difference in survival was reasonable. A trial statistician chimed in to explain that the BaSICS trial did have approximately 89% power for a 10% reduction in mortality (ie, hazard ratio of 0.90), with the assumption of 35% mortality, compared with the observed 27%. The detection of a 1% difference in mortality would require about 100,000 patients and would usually not be considered a clinically significant difference (see Fig 2A for discussion). Although there was some movement of opinions toward normal saline (Fig 2B), many chat participants remained anchored to their biases, especially given the similar costs of normal saline and lactated Ringer’s solutions. The consensus did exist that the quantity of fluids used did matter more than the quality (choice) of which fluid is used, except for hyperchloremic settings (balanced solutions preferred) and traumatic brain injuries (normal saline preferred).

Possibly resolving the question in the fluid wars is the Plasma-Lyte 148 versus Saline trial, a multicenter, randomized controlled trial comparing the effects of Plasma-Lyte 148 versus normal saline solution on mortality among 5,037 critically ill patients that was published soon after the discussion. Similar to the BaSICS trial, this trial reported no difference between the risks of death or AKI among critically ill adults in the ICU with the use of balanced solution compared with normal saline.^11^ An ongoing trial in this area is Better Evidence for Selecting Transplant Fluids, which also compares the effects of Plasma-Lyte 148 versus 0.9% saline on delayed graft function in 800 deceased donor kidney transplants.^12^ It is humbling that almost 200 years after intravenous fluids were invented, we are still struggling to answer fundamental questions regarding their makeup and use. From the evidence so far, outside select settings such as hyperchloremia or traumatic brain injury, the choice of intravenous fluids does not seem to matter.
